# The M-CSF receptor in osteoclasts and beyond

**DOI:** 10.1038/s12276-020-0484-z

**Published:** 2020-08-17

**Authors:** Se Hwan Mun, Peter Sang Uk Park, Kyung-Hyun Park-Min

**Affiliations:** 1 Arthritis and Tissue Degeneration Program, David Z. Rosensweig Genomics Research Center, Hospital for Special Surgery, New York, NY 10021 USA; 2 Department of Medicine, Weill Cornell Medical College, New York, NY 10065 USA; 3 BCMB Allied Program, Weill Cornell Graduate School of Medical Sciences, New York, NY 10021 USA

**Keywords:** Osteoimmunology, Cell signalling, Targeted bone remodelling, Differentiation

## Abstract

Colony-stimulating factor 1 receptor (CSF1R, also known as c-FMS) is a receptor tyrosine kinase. Macrophage colony-stimulating factor (M-CSF) and IL-34 are ligands of CSF1R. CSF1R-mediated signaling is crucial for the survival, function, proliferation, and differentiation of myeloid lineage cells, including osteoclasts, monocytes/macrophages, microglia, Langerhans cells in the skin, and Paneth cells in the intestine. CSF1R also plays an important role in oocytes and trophoblastic cells in the female reproductive tract and in the maintenance and maturation of neural progenitor cells. Given that CSF1R is expressed in a wide range of myeloid cells, altered CSF1R signaling is implicated in inflammatory, neoplastic, and neurodegenerative diseases. Inhibiting CSF1R signaling through an inhibitory anti-CSF1R antibody or small molecule inhibitors that target the kinase activity of CSF1R has thus been a promising therapeutic strategy for those diseases. In this review, we cover the recent progress in our understanding of the various roles of CSF1R in osteoclasts and other myeloid cells, highlighting the therapeutic applications of CSF1R inhibitors in disease conditions.

## Bone disease: Signaling pathway offers promising drug target

Drugs directed at a key signaling receptor involved in breaking down bone tissue could help treat diseases marked by pathological bone loss and destruction. In a review article, Kyung-Hyun Park-Min and colleagues from the Hospital for Special Surgery in New York, USA, discuss the essential roles played by the colony-stimulating factor 1 receptor (CSF1R) protein in the survival, function, proliferation and differentiation of myeloid lineage stem cells in the bone marrow, including bone-resorbing osteoclasts. They explore the links between the CSF1R-mediated signaling pathway and diseases such as cancer and neurodegeneration. The authors largely focus on bone conditions, highlighting mouse studies in which CSF1R-blocking drugs were shown to ameliorate bone loss and inflammatory symptoms in models of arthritis, osteoporosis and metastatic cancer. Clinical trials are ongoing to test therapeutic applications.

## Introduction

Osteoclasts are the only bone-resorbing cells and are differentiated from myeloid lineage precursor cells^1–5^. Osteoclasts play important roles in both homeostatic bone remodeling and pathogenic bone resorption that is associated with inflammatory arthritis, osteoporosis, and bone metastasis in cancer. CSF1R expression is low in immature myeloid precursor cells and increases as the myeloid cells mature^6^. CSF1R-mediated signaling and receptor activator of NF-κB ligand (RANKL) are essential for osteoclast function, proliferation and differentiation from precursor cells^7–11^. CSF1/CSF1R signaling induces the expression of receptor activator of NF-κB (RANK), a receptor for RANKL^12,13^. Upon RANK-RANKL binding, the NF-kB and MAPK signaling pathways are activated to induce MYC and FOS expression, resulting in the induction of metabolic reprogramming and the expression of NFATc1, a master regulator of osteoclastogenesis^14^. NFATc1 drives the osteoclast differentiation program^15,16^. Osteoprotegerin (OPG) is a decoy receptor for RANKL and is secreted from stromal cells and osteoblasts^17^. OPG is a natural inhibitor of RANKL-mediated signaling in osteoclasts by preventing RANK-RANKL interactions and fine-tuning osteoclast differentiation and bone remodeling.

Coordinated regulation between RANKL and costimulatory signals is required for optimal osteoclast differentiation^18^. Costimulatory signals are mediated by DNAX-associated protein 12 kD (DAP12) and FcεR1 gamma chain (FcRγ), which are adaptor molecules containing immunoreceptor tyrosine-based activation motifs (ITAMs), and are also necessary for osteoclastogenesis^19–21^. DAP12 and FcRγ pair and bind with cell-surface receptors and transduce ITAM-mediated signaling. DAP12-associated receptors include TREM2, MDL-1, and siglec-15, while FcRγ-associated receptors are OSCAR, PIR-A, and Fcγ receptors (reviewed in ref. ^18^). DAP12 and FcRγ double-deficient mice exhibit osteopetrosis^20,22^. Upon ligand binding, two tyrosine residues in the ITAM motif are phosphorylated to recruit Syk kinase and activate downstream signaling pathways, which activate PLCγ and calcium signaling to promote RANKL-induced NFATc1 expression. DAP12 is also phosphorylated by CSF1R activation^23^, and crossregulation between DAP12-mediated signaling and CSF1R-mediated signaling in osteoclasts has also been reported^24^.

The essential role of the CSF1/CSF1R axis in osteoclasts has been well established. CSF1R is a type III receptor tyrosine kinase (RTK) that is involved in the proliferation, differentiation, survival, motility, and function of myeloid cells and in promoting disease progression in various conditions ranging from inflammation to cancer^7,25,26^. An autosomal recessive inactivating mutation of the CSF1 gene in op/op mice causes osteopetrosis and other developmental defects that are associated with reduced numbers of osteoclasts and macrophages^27^. The administration of CSF1 to op/op mice rescues the defects in osteoclasts and osteopetrosis^28^. CSF1R-deficient mice largely recapitulate the phenotype of op/op mice and exhibit abnormal skeletal, neural, and glandular development^29^. CSF1R-deficient mice show reduced macrophage and osteoclast numbers and reduced matrix remodeling due to diminished cellular motility and adhesion^26,30^. However, the severity of the osteopetrotic phenotype and systemic depletion of macrophages are much higher in CSF1R-deficient mice than in op/op mice, while the numbers of Langerhans cells and microglia in CSF1R-deficient mice are comparable to those of op/op mice. In op/op mice, hematopoietic deficiencies have been shown to be resolved with age^30^. This discrepancy was explained later by the discovery of interleukin-34 (IL-34), another ligand of CSF1R^31^. Currently, CSF1 and IL-34 are the two known ligands of CSF1R. Both ligands induce osteoclast differentiation^11,32^.

CSF1R also plays an important role in the differentiation of osteoclasts during the developmental period. During embryonic development, macrophage-like cells are produced first in the yolk sac and then appear in the liver^33^. Csf1r mRNA has been detected in the ectoplacental cone early after implantation and in phagocytic cells isolated from the yolk sac^34^. Fate mapping using Csf1r-Mer-iCre-Mer;Rosa26^TdTomato^ mice shows that CSF1R-positive yolk sac macrophages are differentiated from early erythromyeloid progenitors (EMPs) and give rise to neonatal osteoclasts^35^. Conversely, CX_3_CR1^+^ yolk-sac macrophages provide long-lasting osteoclast precursors for postnatal bone remodeling in both physiological and pathological conditions. Osteoclasts originating from EMP-lineage cells are found in fetal ossification centers and are involved in normal bone development and tooth eruption. Postnatal maintenance of osteoclasts comes from the fusion process between long-lived syncytia and hematopoietic stem cell (HSC)-derived circulating cells that express CX_3_CR1^36^. In bone fracture and during homeostasis, circulating CX_3_CR1^+^ osteoclast precursor cells migrate to the bone and become CX_3_CR1^−^TRAP^+^ osteoclasts^37^. Therefore, CSF1R-mediated signals are important for both osteoclast precursor cells and osteoclasts.

Mutations in CSF1R that cause the expression of a mutant receptor or inactivation of one Csf1r allele have been identified in rare neurodegenerative disorders: adult-onset leukodystrophy with axonal spheroids and pigmented glia (ALSP) (also known as hereditary diffuse leukoencephalopathy with axonal spheroids (HDLS)), pigmented orthochromatic leukodystrophy (POLD), and pediatric-onset leukoencephalopathy^38–41^. Inactivation of one allele of Csf1r is sufficient to cause ALSP in a mouse model^42^ with some differences in neuropathological findings^43^. In addition, circulating slan^+^CD14^+^CD16^+^ monocytes that express high levels of CSF1R are depleted in patients with HDLS^44^. In inflammatory brain disorders, circulating monocytes might enter and repopulate the brain, although microglia originate from the yolk sac^45^. While HDLS patients with monoallelic CSF1R mutations do not exhibit osteopetrosis or bone abnormalities^38^, biallelic CSF1R mutations cause skeletal disorders and osteosclerosis^41,46^. Patient studies have established the important role of CSF1R in the regulation of not only microglia in the brain but also CSF1R-sensitive blood myeloid cells. Analyses of bone cells and bone phenotypes in patients with CSF1R mutations need to be further conducted to establish the link between osteoclasts and CSF1R-mediated signaling in humans.

In this review, we describe the basic aspects of CSF1R, the function of CSF1R in osteoclasts, and the effect of pharmacological inhibition of CSF1R on disease progression in preclinical and clinical settings.

## The structure of CSF1R

### The CSF1R gene

**Fig. 1:**
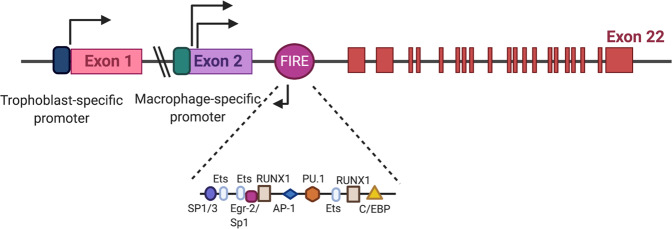
Genomic structure of the mouse Csf1r locus. Both human and mouse Csf1r genes consist of 22 exons and 21 introns (upper panel). Exon 1 is only expressed in trophoblasts through activation of the trophoblast-specific promoter. The human trophoblast-specific promoter is located 20 kb upstream of exon 1. Csf1r transcription in macrophages starts from the promoter that is upstream of exon 2. Neither promoter has a TATA box. The Fms-intronic regulatory element (FIRE) is a highly conserved regulatory element that produces antisense transcripts to overcome the unknown repressive elements in intron 2. Several transcription factors known to bind to the FIRE have been characterized (lower panel).

Only myeloid cells express *Csf1r* mRNA (Fig. 1: the murine Csf1r gene). The Csf1r gene is located on human chromosome 5 (5q32)^47^ and in a syntonic region on mouse chromosome 18 (18D)^48^. The Csf1r gene consists of 21 introns and 22 exons. The expression of the Csf1r gene is mediated by two alternative promoters and occurs in a tissue-specific manner. The first exon of Csf1r is transcribed only in trophoblasts, and the second exon of Csf1r is transcribed in macrophages. Deletion of the trophoblast-specific promoter regions in the Csf1r-EGFP transgenic line also abolishes the expression of EGFP in osteoclasts^49^. The transcriptional activation of Csf1r involves many transcription factors, including Ets (the E26 transformation-specific family of transcription factors), PU.1, ATF, C/EBP, RUX, AP-1, IRF, STAT, KLF, REL, and FUS/TLS^50^. The Csf1r promoter is filled with multiple PU.1/Ets binding sites around the transcription start site. However, the proximal CSF1R promoter lacks a TATA box and other classic promoter elements. It has been suggested that a loose repeat of CAG or CAA immediately adjacent to the dominant start site bound by Ewing sarcoma (EWS) and FUS/TLS, which are two TATA-associated factors, might substitute for the TATA-binding protein in macrophages^51^.

The expression of the Csf1r gene is regulated by two highly conserved regions: the promoter upstream of exon 2 and the fms intronic regulatory element (FIRE). Yue et al. show that in macrophages, a 3.5 kb exon 2 promoter facilitates the maximal expression of Csf1r and further suggest that the 0.3 kb promoter is as active as the 3.5 kb promoter^52^. Moreover, the FIRE is a 250-bp region in intron 2^53^. The FIRE is an important regulatory element of the CSF1R gene in macrophages and controls transcript elongation during macrophage-specific transcription of CSF1R^54^. The FIRE contains the consensus binding sites for many transcription factors (Fig. 1)^55^. The FIRE also encodes antisense transcripts that start from two antisense transcription start sites^6^. Inverting the orientation of the FIRE diminishes its enhancer activity in macrophages^54^, suggesting that the FIRE is an orientation-specific transcriptional enhancer element. An antisense transcript encoded by the FIRE may contribute to its ability to overcome repression by uncharacterized repressive elements within intron 2. During the differentiation of macrophages from immature precursor cells, the recruitment of transcription factors and chromatin remodeling occur first in the proximal Csf1r promoter and then in the FIRE, allowing only differentiated macrophages to express higher levels of Csf1r. However, a recent study shows that the genomic deletion of the FIRE in mice selectively impacts CSF1R expression and demonstrates the functional importance of the FIRE only in specific macrophage populations^56^. Ablation of the FIRE in mice depletes embryonic macrophages in embryo and tissue macrophages, including microglia in the brain and resident macrophages in the skin, kidney, heart, and peritoneum^56^. In contrast to CSF1R-deficient mice, FIRE-deficient mice are healthy and fertile without growth, neurological, or developmental abnormalities such as osteopetrosis and failure of tooth eruption, suggesting that the FIRE is not required for Csf1r expression in all types of myeloid cells.

### The CSF1R protein

**Fig. 2:**
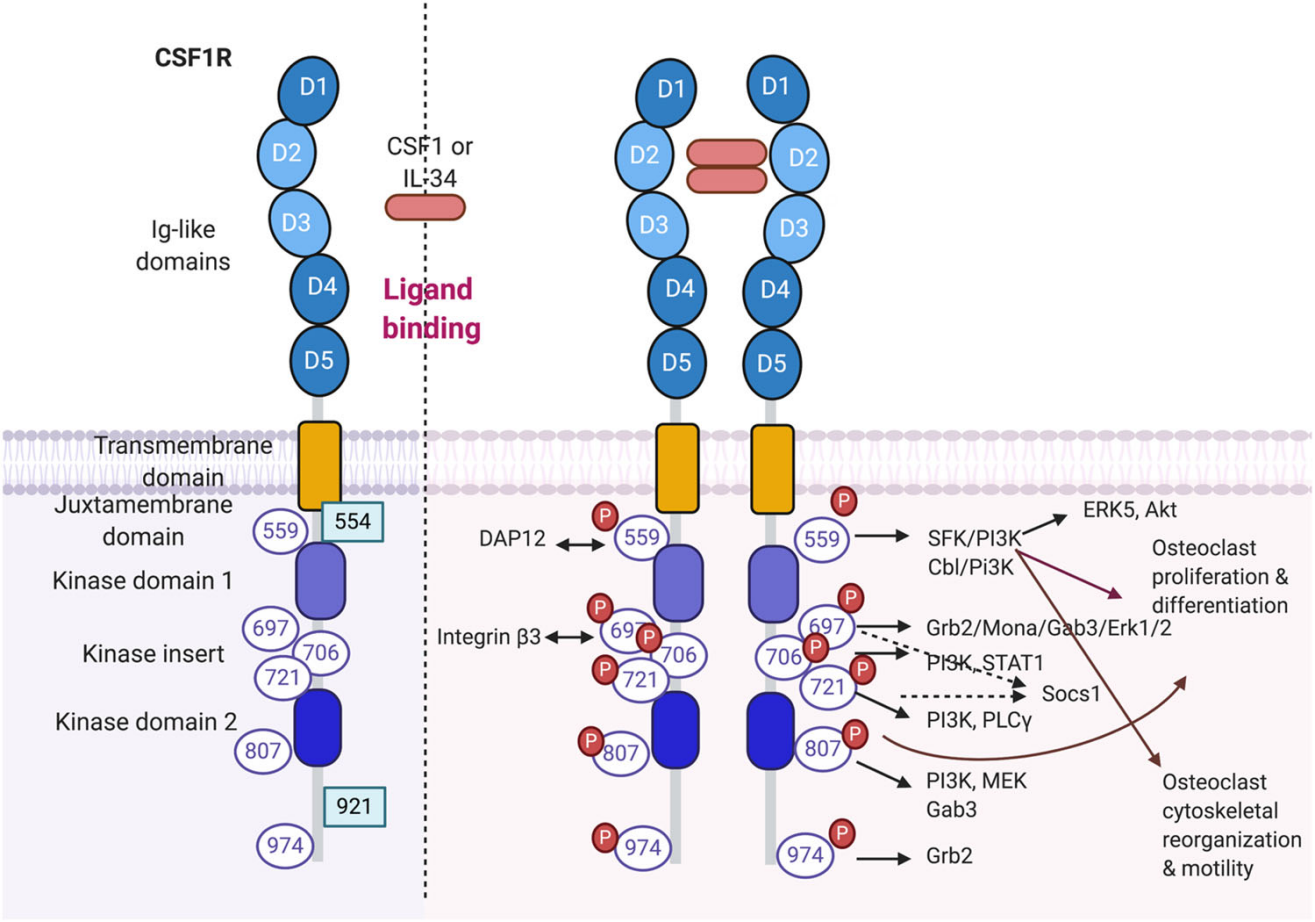
Structure of CSF1R protein. The left panel shows the structure of CSF1R. The extracellular regions have five Ig-like domains; D2 and D3 are ligand binding domains. The intracellular domains consist of the transmembrane domain, the juxtamembrane domain, two-kinase domains, a kinase insert, and cytoplasmic domains. CSF1R is a receptor tyrosine kinase and has 6 tyrosine residues that are phosphorylated upon ligand binding (purple circle). Green square: phosphorylated tyrosine in the v-FMS oncogenic receptor. Ligand engagement of CSF1R induces dimerization and phosphorylation of tyrosine residues. Phosphorylated tyrosines serve as docking sites for Src homology domain 2-containing signaling molecules to promote osteoclast differentiation, proliferation, cytoskeletal reorganization, and motility.

The structures of human CSF1R and mouse CSF1R are highly conserved. CSF1R is divided into two parts: the extracellular domain and the intracellular cytoplasmic domain (Fig. 2)^57,58^. The extracellular domain contains immunoglobulin (Ig)-like domains to which ligands bind, a linker region, and a single-pass transmembrane helix. Three N-terminal Ig domains (D1–D3) contribute to ligand recognition, while the next two Ig domains (D4–D5) are involved in stabilizing the ligand-receptor complex. The cytoplasmic domain consists of two-kinase domains, a kinase insert, a juxtamembrane domain, and a carboxyterminal tail. CSF1R also undergoes posttranslational modifications such as phosphorylation and glycosylation. In the absence of ligands, CSF1R is in an inactive autoinhibitory state. Upon ligand binding, the juxtamembrane domain moves from the autoinhibitory position, and CSF1R shifts to an activated, extended conformation^59^.

CSF1R-expressing cells have been identified using CSF1R-EGFP transgenic mice^60,61^. The regulatory elements of the murine Csf1r locus, including 150 bp of the distal promoter, have been used to generate CSF1R-EGFP mice. EGFP expression is detected in placental trophoblasts from the earliest stage of implantation at embryonic day (ED) 7.5, as well as in various stages of development^58^. During embryonic development, EGFP expression is also detected in cells from the yolk sac at ED 8.5–9, in the head of embryo at ED 9.5, in the dorsal midline at ED 10.5, in the skin at ED12.5, and in F4/80-positive cells at ED 11–12. Csf1r-mApple transgenic mice also exhibit a similar pattern of expression as Csf1r-EGFP mice^62^. CSF1R expression begins in the early embryonic developmental stage in oocytes and in preimplantation embryos^63,64^. CSF1R protein expression is restricted to myeloid cells and is much higher in tissue resident macrophages than in blood monocytes.

## CSF1R signal transduction pathways

CSF1R signal transduction pathways play key roles in the survival, differentiation, and functions of myeloid cells and have been extensively studied in macrophages, osteoclasts, and microglia (reviewed in ref. ^58^).

### CSF1R signaling pathways

Ligand binding leads to rapid dimerization and autophosphorylation of CSF1R (Fig. 2). Six tyrosine residues in the CSF1R cytoplasmic domain (Y559, Y697, Y706, Y721, Y807, and Y974) and two tyrosine residues in an oncogenic form of Csf1r (Y544 and Y921) have been described as being phosphorylated^65,66^. Most of these phosphorylated tyrosines serve as docking sites for adaptor proteins containing the Src homology 2 (SH2) domain, and these adaptor proteins further relay downstream signaling events. The function of each tyrosine residue is determined based on mutagenesis experiments. Upon ligand binding, Tyr559 in mouse CSF1R or Tyr561 in human CSF1R is the first phosphorylated tyrosine, providing a docking site for Src family kinase (SFK)/Cbl and regulating ERK5 and Akt activity. Cbl is an E3 ubiquitin kinase^67^, and activation of SFK/Cbl leads to the multiubiquitination of CSF1R and receptor internalization and degradation^68^. Phosphorylated Cbl also recruits and stabilizes multiprotein complexes. Tyr559 maintains CSF1R inactivity in the absence of ligands^66^. Ligand-induced phosphorylation of Tyr559 releases autoinhibition and permits full activation of CSF1R in macrophages^69^. Tyr559 plays a critical role in CSF1R activation, cell proliferation, intracellular signaling, and cell death of macrophages relative to those of Tyr697 and Tyr807^70^. In addition, SFK connects to the DAP12 adaptor protein, which is also phosphorylated by CSF1R activation^58^. DAP12 is required for CSF1-induced phosphorylation and stabilization of β-catenin^23^. DAP12 deficiency impairs CSF1R-mediated macrophage proliferation and survival. Tyr697, Tyr706, and Tyr721 are located in the kinase insert domain. Tyr697 is a docking site for growth factor receptor-bound protein-2 (Grb2)^71^. Grb2 is involved in CSF1R-mediated ERK activation through the nucleotide exchange factor Sos^72^. Mona is an adaptor protein that is induced by CSF1. Mona has one SH2 domain and two SH3 domains and binds to Grb2 via Tyr697 in CSF1R^73^. After the first wave of tyrosine phosphorylation, including the transient formation of the CSF1R/Grb2/Sos complex, the second wave of tyrosine phosphorylation is accompanied by serine phosphorylation and cbl-dependent CSF1R ubiquitination^74^. Tyr706 is associated with phosphatidylinositol 3 (PI3)-kinase^75^ and mediates CSF1-induced STAT1 activation^76^. Tyr706 also suppresses the expression of CD11b in macrophages^77^. Tyr721 is the binding site for PI3K and PLCγ, which leads to macrophage differentiation^78^. Tyr807 and Tyr921 are in the carboxyterminal tail of CSF1R. Tyr807 is conserved throughout all protein tyrosine kinases and is required for macrophage differentiation^79^. The v-FMS oncogene of feline sarcoma virus encodes an oncogenic FMS protein that differs from CSF1R in only seven amino acids and in the C-terminal region. Two additional tyrosines in v-FMS are phosphorylated. Tyr543/544 is located in the juxtamembrane domain and is associated with the p55 polypeptide^80^. In addition to Tyr696, Tyr921 in the vFMS oncogene also serves as the second docking site for Grb2^65^.

In addition to ligand-induced tyrosine phosphorylation, the ligand-CSF1R complex is internalized by endocytosis, which quickly terminates CSF1R signaling and leads to the degradation of CSF1R and CSF1^81,82^. In addition, Huynh et al. reported that internalized CSF1R mediates sustained ERK1/2 and Akt signaling^83^. Early CSF1R signaling (~2 h) also induces increased protein synthesis and regulates macrophage protein turnover^84^. In addition, ligand-induced ectodomain shedding may regulate CSF1R activity. CSF1R signaling pathways also regulate macrophage morphology, adhesion, and motility^85^. Ablation of CSF1R leads to macrophage rounding by increasing the level of protein tyrosine phosphatase phi/paxillin/Pky2 complexes^86^.

### CSF1R signaling in osteoclasts

CSF1R-mediated signaling plays important roles in the differentiation of osteoclast precursor cells and mature osteoclasts. Csf1r-deficient mice or op/op mice have a severe osteoclast deficiency and weak long bones that show a disorganized matrix, reduced mineralization, and abnormal osteoblasts^27,87^. During in vitro osteoclastogenesis, CSF1R-mediated signaling induces RANK expression in osteoclast precursor cells. The role of tyrosine phosphorylation of CSF1R in osteoclasts has been investigated using Epo receptor/CSF1R chimeras^88^. In the experiment, each tyrosine in the Epo/CSF1R receptor is mutated to phenylalanine. When stimulated with Epo and RANKL, cells expressing the Y559F mutant receptor fail to differentiate into osteoclasts, while those expressing either Y559F or Y807F fail to resorb bone. This study shows that Tyr559 and Tyr807 in CSF1R are essential for osteoclast proliferation and differentiation, whereas Tyr697, Tyr706, and Tyr721 exert no effects on osteoclasts^88^. Cbl is able to bind to phosphorylated Tyr559. In osteoclasts, Cbl-PI3K does not affect CSF1R-mediated proliferation and differentiation of precursors but is required for survival and actin reorganization in mature osteoclasts^89^.

In addition to the differentiation and proliferation of osteoclasts, CSF1R-mediated signaling stimulates motility and regulates cytoskeletal reorganization in a c-Src-dependent manner via Tyr559^90^. Crosstalk between CSF1R and other important molecules in osteoclasts, including integrin β3 and DAP12, regulates cytoskeletal reorganization and adhesion of mature osteoclasts. Integrin β3 is induced by RANKL stimulation and binds to extracellular matrix proteins such as vitronectin, osteopontin, and bone sialoprotein^91^. Integrin β3-deficient mice have dysfunctional osteoclasts and develop an osteosclerotic phenotype^91–93^. However, in vitro osteoclastogenesis of integrin β3-deficient cells is blunted, and enhanced CSF1/CSF1R-mediated signaling rescues the defective osteoclasts in integrin β3-deficient mice^94^. High-dose CSF1 treatment promotes prolonged ERK activation and c-FOS expression in integrin β3-deficient mice mediated by Tyr697 in CSF1R. DAP12 is an adaptor molecule containing an immunoreceptor tyrosine-based activation motif (ITAM) and is necessary for osteoclastogenesis^19–21^. Activation of DAP12-associated receptors induces the phosphorylation of ITAM by Src family kinases and recruits Syk or Zap70 tyrosine kinases, which regulate cytoskeletal remodeling in osteoclasts. DAP12 is activated by CSF1R-mediated signaling and is involved in CSF1R-mediated cytoskeletal remodeling in osteoclasts^24^. Tyr559 is necessary for the activation of DAP12/syk-mediated signaling. A high dose of CSF1 also partially rescues the defects in osteoclastogenesis in DAP12-deficient cells^21^. In addition, it has been shown that pretreatment with inflammatory signals such as LPS and IL-1 prior to RANKL stimulation suppresses osteoclastogenesis in part by inducing ectodomain shedding and CSF1R degradation^95,96^. In both physiological and pathological conditions, CSF1R-mediated signaling contributes to osteoclast differentiation and activity. However, the downstream signaling networks of CSF1R in osteoclasts are an area of research that needs further investigation.

## CSF1R ligands and their differential functions in CSF1R signaling

CSF1R binds to two different ligands: CSF1 and interleukin-34 (IL-34)^31^. Although these ligands share the same receptor, there are structural, functional, and spatial differences between CSF1 and IL-34. IL‐34 has no apparent consensus structural domain or motif and shares no sequence similarity with CSF1^97^. The CSF1/CSF1R complex and the IL-34/CSF1R complex also do not share structural similarities^97^. The CSF1/CSF1R complex has hydrophilic interactions, while the IL‐34/CSF1R complex contains a large number of hydrophobic regions. While CSF1 is detectable in circulation, IL-34 is not circulated in blood. Although CSF1R is only receptor for CSF1, IL-34 has been shown to bind to the extracellular domains of CSF‐1R, receptor‐type protein‐tyrosine phosphatase‐zeta (PTP‐ζ), and the chondroitin sulfate chains of syndecan‐1^98,99^. Although IL-34 and CSF1 have equivalent ability to induce macrophage differentiation with comparable kinetics, CSF1 and IL-34 have different capability for macrophage polarization. IL-34-derived M1 and M2 macrophages have significantly higher secretion of IL-10 and CCL17, respectively, than their CSF1-derived counterparts^100^. Furthermore, macrophages differentiated from human peripheral blood monocytes with IL-34 exhibit greater resistance to HIV-1 infection than those derived from CSF1 due to increased expression of pertinent restriction factor genes^101^. IL-34 has also been demonstrated to repolarize rat M1 Kupffer cells (KCs) in the liver to the M2 phenotype by activating the PI3K/protein kinase B/mammalian target of rapamycin (mTOR) pathway and could be used therapeutically to reduce acute rejection during liver transplantation^102^. Each of these ligands might play a different role in osteoclasts and other myeloid cells, which are discussed below.

### CSF1

CSF1 (also known as macrophage colony-stimulating factor (M-CSF)) is a ligand for CSF1R and a growth factor that regulates the survival, proliferation, and differentiation of cells of hematopoietic lineages (reviewed in refs. ^103,104^). The CSF1 gene is located at chromosome 1 p21-p13 in humans and at chromosome 3, 51 cM in mice^105^. CSF1 exists as a membrane-bound protein, a secreted proteoglycan, and a secreted glycoprotein. Furthermore, CSF1 has several isoforms. Short, membrane-bound CSF1 is generated from 1.6 and 3.1 kb transcripts, and 2.6, 3.7, and 4 kb transcripts generate the secreted forms of M-CSF. CSF1 also forms a homodimer^106^. CSF1 is widely expressed in many cell types, including osteoblasts, stromal cells, fibroblasts, epithelial cells, and several metastatic tumor cells^107–110^. CSF1 is secreted as a glycoprotein or proteoglycan^111,112^ or is expressed as a cell-surface protein on the cell membrane^113^. It has been established that cell-surface CSF1 and secreted CSF1 differentially regulate osteoclastic bone resorption. CSF1 alone can correct osteoclast deficiency in CSF1-deficient op/op mice^114^. Although it has been shown that membrane CSF1 is important for osteoblast-mediated osteoclastogenesis in the bone microenvironment^115–118^, transgenic op/op mice expressing membrane-spanning cell-surface CSF1 cannot fully recover from osteopetrosis and hematologic abnormalities in the blood and bone marrow and show delayed trabecular bone resorption^119^.

Systemic injection of CSF1 exacerbates the symptoms of collagen-induced arthritis (CIA) and acute-induced arthritis, suggesting the involvement of CSF1 in inflammatory arthritis by increasing myeloid cells^120,121^. In an inflammatory osteolysis model, TNFα induces CSF1 production in stromal cells, and CSF1 contributes to increased osteoclastogenesis^122^. In several pathological bone diseases, including osteoporosis and inflammatory arthritis, a correlation has been observed between an increased number of osteoclasts and elevated levels of CSF1^123–126^. In addition to enhancing inflammatory responses^127^, CSF1 also plays a role in pain development associated with inflammatory arthritis^128,129^. However, the exact underlying mechanism of CSF1 in inflammatory arthritis is not completely understood. Estrogen suppresses the mRNA and protein expression of CSF1 by controlling Egr-1 and Sp-1^130,131^. In an estrogen-deficient state, increased Sp-1 binds to the Csf1 gene and enhances CSF1 expression. In addition, elevated serum CSF1 has been detected in patients with breast cancer and have been correlated with an adverse prognosis^132,133^. As such, CSF1 is considered a potential prognostic marker for patient survival and disease recurrence^134^.

### IL-34

IL-34 is a cytokine that binds to CSF1R and is synthesized as a secreted glycoprotein^31,135^. Similar to CSF1, IL-34 activates downstream signaling pathways and regulates major cellular functions, including proliferation, differentiation, survival, metabolism, cellular adhesion, migration, and cytokine/chemokine expression. IL‐34 exists in all vertebrates, including fish, amphibians, birds, and mammals and exhibits high conservation among species. Structurally, IL‐34 belongs to the short‐chain helical hematopoietic cytokine family but shows no apparent consensus structural domains, motifs, or sequence homology with other cytokines. The IL-34 gene is located in humans on chromosome 16q22.1 and in mice on chromosome 8E1^31^. In the steady state, IL‐34 contributes to the development and maintenance of specific myeloid cell subsets in a tissue‐specific manner: Langerhans cells in the skin and microglia in the brain^63,136,137^. IL-34 has important roles in the differentiation of Langerhans cells (LCs) and microglia in the gray matter^138,139^. IL-34 is also present at the fetal-maternal interface and plays a key role in the polarization of macrophages to a decidual phenotype^140^. Furthermore, IL-34 is essential for the movement of yolk sac macrophages to the embryonic brain and the earliest seeding of the brain by microglia^141^. Brain-derived IL-34 is responsible for the migration of microglial precursors to the proximal brain region^142^. Finally, IL-34 secreted from the follicular dendritic cell line FL-Y was found to induce the differentiation of a new type of monocytic cell called follicular dendritic cell-induced monocytic cells by associating with the molecular chaperone 78-kDa glucose regulated protein^143^.

In addition to its function in mammals, IL-34 is required in aquatic organisms as well. IL-34 homologs from mudskippers (*Boleophthalmus pectinirostris*) play key roles in the differentiation of mudskipper monocytes and macrophages into proinflammatory phenotypes^144^. In zebrafish, ectopic expression of IL-34 stimulates the in vivo enrichment of macrophages in the liver^145^. In one frog species (*Xenopus laevis*), IL-34-derived macrophages have a higher expression of pattern recognition receptor (PRR) genes related to viral and bacterial pathogen-associated molecular pattern (PAMP) recognition than their CSF1-driven counterparts^146^.

IL-34 is a novel regulator of osteoclastogenesis and plays a key role in pathological bone destruction by driving the onset and development of inflammatory arthritis. IL-34 induces osteoclast formation in human CD14+ monocytes^147^. IL-34 promotes the proliferation and differentiation of murine bone marrow-derived macrophages (BMMs) into osteoclasts in lieu of CSF1^32^. Furthermore, IL-34 alone provides a sufficient signal for BMM survival, and in combination with RANKL, it increases the expression of p-STAT3. Treatment with AG490, an inhibitor of JAK2/STAT3 signaling, reduced both IL-34- and RANKL-driven osteoclastogenesis and increased Smad7 expression, while the inhibitor had a negligible effect on osteoclastogenesis induced by M-CSF and RANKL^148^. These results suggest that IL-34 may regulate osteoclastogenesis in part by increasing p-STAT3 and decreasing Smad7. Furthermore, IL-34 is expressed in the mouse multiple myeloma (MM) cell line MOPC315. BM supports osteoclastogenesis in vitro and accelerates MM-induced osteolysis in vivo.

In pathological conditions, changes in IL‐34 expression are correlated with disease progression, severity, and chronicity^149^. Due to its osteoclastogenic effect, IL-34 is continuously being investigated as a potential diagnostic marker for inflammatory bone and joint diseases such as rheumatoid arthritis (RA). Plasma levels of IL-34 and RANKL in patients with RA are significantly higher than those of healthy controls. Furthermore, there is a significant correlation between IL-34 and bone erosion, as measured by ultrasound^150^. Similarly, serum levels of IL-34 in patients with psoriatic arthritis are significantly higher than those in patients with psoriasis alone or healthy controls^151^. Mechanistically, it has been shown that IL-34 could promote rheumatoid fibroblast-like synoviocytes (FLS) to produce IL-6, which increased the level of Th17 and further advanced RA^152^. Furthermore, IL-34 levels in gingival crevicular fluid (GCF) of patients with either chronic or aggressive periodontitis were higher than those of healthy controls^153^. Finally, IL-34 levels in the serum and synovial fluid of patients with osteoarthritis (OA) correlated with the radiographic and symptomatic severity of OA^154^.

In addition, IL-34 shows promise as a diagnostic marker for diverse inflammatory conditions, including liver fibrosis, atherosclerosis, Hashimoto’s thyroiditis, and lupus nephritis. The serum level of IL-34 is related to inflammatory activity in the liver and is highly sensitive to severe liver fibrosis in patients with chronic hepatitis B virus infection^155^. IL-34 treatment increases the formation of foam cells by upregulating CD36 expression through the p38-MAPK signaling pathway in bone marrow-derived macrophages, suggesting its role in promoting the development of atherosclerosis^156^. In patients with Hashimoto’s thyroiditis, the serum level of IL-34 was significantly less than that of healthy controls and could be evaluated to measure thyrocyte damage^157^. Moreover, patients with lupus nephritis (LN) have higher levels of IL-34 in the serum than healthy controls^158^. A study using MRL-Faslpr mice, a mouse model of lupus, revealed that mice lacking IL-34 exhibit decreased nephritic symptoms. In mice, IL-34 causes intrarenal macrophages to accumulate by inducing monocyte proliferation in the bone marrow and stimulates tubular epithelial cells to undergo apoptosis^159^. A study using human mesangial cells (HMCs) discovers that IL-34 is highly expressed in the HMCs of LN patients and is suppressed by treatment with DDK1, an inhibitor of the Wnt pathway^160^.

## Diseases and therapeutic applications of CSF1R inhibition

CSF1R-mediated signaling plays an important role in many diseases, including inflammatory arthritis, neurodegenerative diseases, Alzheimer’s disease, cancer, atherosclerosis, lung fibrosis, diabetes, multiple sclerosis, and systemic lupus erythematous^161^. Myeloid cells have been found to promote disease progression, and either blocking the differentiation or recruitment of myeloid cells is considered a potential strategy to attenuate disease conditions. Various approaches targeting either CSF1R or its ligands are currently in clinical development (Table 1). In addition, CSF1R inhibition has been relatively well tolerated and demonstrates good specificity. CSF1R targeting has shown beneficial effects in models of cancer metastasis and inflammatory diseases although deleterious effects have been also observed in colitis^162^ and skeletal muscle regeneration^163^. Thus, the use of CSF1R inhibitors or antibodies might be a promising strategy for treating myeloid cell-dominant diseases. We discuss the potential therapeutic effects of targeting CSF1R or its ligands on bone diseases, neurological disease, and cancer.

**Table 1:** Therapeutic applications of inhibitors and antibodies against CSF1R.

Name	Form	Targets	Function	Clinical Trial diseases	Reference
Pexidartinib (PLX3397)	Small molecular inhibitor	CSF1R, c-KIT, VEGFR, and Flt3	Inhibition of CSF1R signaling	Autoimmune diseases, cancer, and Alzheimer’s disease	^172,193,195,198,204,206^
Imatinib	Small molecular inhibitor	CSF1R, ABL, c-KIT, and PDGFR-β	Inhibition of CSF1R kinase activity	Osteoporosis, osteolysis, chronic myeloid leukemia (CML), and breast cancer	^153,168,169^
PLX5622	Small molecular inhibitor	CSF1R	Inhibition of CSF1R signaling	Rheumatoid arthritis, cancer, neuropathic pain, and Alzheimer’s disease	^187–189,191,194,196^
BLZ945	Small molecular inhibitor	CSF1R, c-Kit, PDGFRβ, and Flt3	Inhibition of CSF1R signaling	Cancer and amyotrophic lateral sclerosis	^197,207,208^
GW2580	Small molecular inhibitor	CSF1R	Inhibition of CSF1R kinase activity	Arthritis, osteoporosis, and cancer	^165,190,203,210–212^
Ki20227	Small molecular inhibitor	CSF1R, VEGFR2, c-KIT, and PDGFRβ	Inhibition of CSF1R kinase activity	Osteolytic bone destruction and breast cancer	^164,183^
Edicotinib (JNJ-40346527)	Small molecular inhibitor	CSF1R, KIT, and Flt3	Inhibition of CSF1R signaling	Alzheimer’s disease, rheumatoid arthritis, and neurodegenerative diseases	^173,174,186^
AFS98 (anti-mouse CSF1R)	Monoclonal antibody	CSF1R	Blockade of CSF1R	Cancer, arthritis, and diabetic nephropathy	^167,217,218^
M279 (anti-mouse CSF1R)	Monoclonal antibody	CSF1R	Blockade of CSF1R	Cancer, arthritis, and bone loss	^177^

### Bone diseases

In the field of bone research, the suitability of targeting CSF1R in treating bone diseases is continually being investigated. Blockade or depletion of CSF1R suppresses the formation and activity of osteoclasts and attenuates pathological bone resorption in inflammatory arthritis, inflammatory bone destruction, and osteoporosis. Serum IL-34 and CSF1 are linked to the disease activity in patients with rheumatoid arthritis and osteoporosis. Blocking the activation of CSF1R by CSF1R inhibitors such as Ki20227^164^ and GW2580^165^ or by antibodies such as anti-CSF1 antibodies^166^ and AFS98^167^ attenuates the progression of joint inflammation, bone erosion, and systemic bone erosion in animal models of arthritis. Administration of anti-CSF1R antibodies suppresses osteoclastogenesis and bone resorption in both serum-induced inflammatory arthritis and TNFα-induced inflammatory ostelysis^122^. Imatinib, a tyrosine kinase inhibitor, prevents and treats arthritis induced by type II collagen antibody (CAIA) and collagen-induced arthritis^153,168^. Imatinib also suppresses CSF1R expression^169^ and enhances mature osteoclast apoptosis. Imatinib decreases the proliferation of rheumatoid arthritis synovial cells, suggesting that CSF1R inhibition could potentially mitigate rheumatoid arthritis-induced bone damage^170,171^. The CSF1R inhibitor PLX3397 significantly inhibits lipopolysaccharide (LPS)-induced bone erosion and the reduction in biomechanical properties in a rat model^172^. Injection of CSF1R neutralizing antibodies, which were previously shown to inhibit LPS-induced osteoclastogenesis in vivo, reduces orthodontic relapse in mouse models^173^. However, the administration of JNJ-40346527 to active rheumatoid arthritis patients for 12 weeks does not show any efficacy, despite the increase in the levels of circulating CSF1^174^. The effect of JNJ-40346527 on bone erosion in the same clinical trial remains unknown. In addition, the effect of small molecules that regulate CSF1R degradation or expression on inflammatory arthritis has also been investigated. Proteasome inhibitors such as MG132 and bortezomib, which can inhibit osteoclast differentiation, also ameliorate LPS-induced bone degradation in mice potentially by accelerating the degradation of CSF1R^175^. Downregulation of CSF1R and receptor activator of NF-kB (RANK) using extracellular binding immunoglobulin protein (BiP) reduces inflammation and bone loss in the human tumor necrosis factor transgenic (hTNFtg) mouse model^176^.

CSF1R-mediated signaling contributes to the pathophysiology of osteoporosis, and recent studies show its potential applicability in treating bone diseases such as osteoporosis. Systemic administration of anti-CSF1R antibodies ablates osteoclasts, increases bone density, and prevents mice from age-induced bone loss^177^. Anti-CSF1R antibodies also deplete tissue macrophages from many different organs and further block the replenishment of macrophages^178^. Neutralizing CSF1 in vivo completely protects mice from ovariectomy (OVX)-induced bone loss^179^. Selective deletion of the double isoform of CSF1 also ameliorates OVX-induced bone loss in mice^180^. Moreover, a newly developed bispecific inhibitor of CSF1R and αvβ3 integrin specifically inhibits osteoclast activity in vitro and reduces serum CTX-1 in ovariectomized mice^181^. In addition, CSF1R-mediated signaling has been shown to mediate osteolysis in metastatic tumors. Serum CSF1 is increased in patients with lung cancer, and knockdown of CSF1 reduces osteoclasts and improves bone metastasis^182^. Gorham‐Stout disease (GSD) is a rare bone disease characterized by massive osteolysis associated with elevated levels of CSF1 produced by lymphatic endothelial cells (LECs), and the CSF1R inhibitor Ki20227 suppresses bone destruction in LEC-induced osteolysis^183^. Although the effect of CSF1R inhibitors on cancer has been extensively explored and is described below, their effect on osteolysis of metastatic tumors has not been well documented.

### Neurological diseases

In the brain, CSF1R is abundant in microglia and other glial cells. Microglia have myriad roles in the nervous system and are consequently studied for their potential use in a wide variety of neurological conditions, ranging from Alzheimer’s disease to epilepsy^184^. Microglia are dependent on CSF1R for survival and proliferation^185^. CSF1R inhibitors have also been used to examine the roles of microglia during neurological development and healing processes. Intriguingly, anti-IL-34 and anti-CSF1 differentially deplete microglia in the gray and white matter of the brain^139^, and CSF1R inhibitors such as JNJ-40346527 attenuate microglial proliferation^186^. Elimination of embryonic microglia with PLX5622 increased weight gain, reduced the number of pro-opiomelanocortin (POMC) neurons, resulted in abnormal cranial and dental formation, and exhibited long-term sex-specific effects, suggesting that microglia could play crucial roles in the early development of the hypothalamus^187^. Moreover, inhibition of microglia with low doses of PLX5622 or long-term oral treatment did not significantly affect the number of oligodendrocyte progenitor cells (OPCs) ex vivo and in vivo in mouse models, indicating that microglia may not be essential for OPC viability^188^. Moreover, depletion of microglia during laser-induced choroidal neovascularization (CNV) in PLX5622-treated mice accelerated the ablation of CNV lesion size and decreased macrophage accumulation in the laser site^189^. Similarly, chronic CSF1R inhibition with GW2580 reduced spinal cord injury-induced microglial/macrophage proliferation in mice and improved the recovery of fine motor control^190^. Finally, PLX5622 significantly mitigated neuropathic pain in mice induced by partial sciatic nerve ligation by reducing the accumulation of macrophages in the damaged nerves, inhibiting CD86+ M1-like macrophages, and attenuating the expression of proinflammatory cytokines such as TNF-α^191^.

Increased proinflammatory cytokine levels and increased CSF1 have been found in Parkinson’s disease (PD) patients^192^. CSF1R inhibitors can deplete microglia. Early long-term PLX3397 treatment of 5XFAD mice, which demonstrates significant neuronal loss similar to that of Alzheimer’s disease, significantly decreases amyloid accumulation and neuritic plaque disposition by decreasing microglial cells^193^. The newly synthesized brain-penetrating CSF1R inhibitor PLX5622 also decreases parenchymal plaque formation in a 5XFAD mouse model by inducing long-term microglial depletion^194^. Similarly, treatment with PLX3397 causes microglial depletion and consequently improves sensory motor functions and depressive-like behavior in mice that were administered 6-hydroxydopamine, which induces pathological symptoms of Parkinson’s disease such as the destruction of dopaminergic neurons^195^. Administration of PLX5622 in an experimental murine autoimmune encephalomyelitis model of multiple sclerosis (MS) attenuates the microglial population and significantly decreases the symptoms of MS, such as demyelination^196^. Treatment with the CSF1R kinase inhibitor BLZ945 also increased remyelination specifically in the striatum/cortex and decreased demyelination in the corpus callosum in the murine cuprizone demyelination model by ablating microglia and increasing oligodendrocytes^197^. Moreover, CSF1R inhibition by PLX3397 in epileptic mice significantly reduces the frequency of seizures by affecting module 18 expression and macrophage phenotype without depleting microglial cells^198^.

### Cancer

Aberrant expression of CSF1R contributes to the development of cancer, including Hodgkin lymphoma and anaplastic large cell lymphoma^199^. Increasing evidence supports that CSF1R affects tumor-associated macrophages (TAMs) to promote tumor progression. TAMs are alternatively activated macrophages that infiltrate into tumors and exhibit anti-inflammatory and protumorigenic phenotypes. TAMs contribute to tumor progression at multiple levels. Blocking CSF1R inhibits the accumulation of immunosuppressive TAMs. Metastasis and TAM infiltration of spontaneous MMTV-PyMT breast tumors are delayed in CSF1R-deficient op/op mice, providing the first evidence for the effect of CSF1R on TAMs^200^. Moreover, CSF1R-positive macrophages correlate with poor survival of patients with lymphoma and solid tumors^201,202^. A study using GW2580 shows that inhibiting CSF1R could impair the progression of hepatocellular carcinoma in mice^203^. While it has been demonstrated that inhibiting CSF1R via PLX3397 (pexidartinib)^204^ has antitumor effects in adult T-cell leukemia/lymphoma (ATLL) by inducing ATL-T cells to undergo apoptosis and reducing the expression of PD-L1/L2^205^, another study shows that PLX3397 induces pancreatic ductal adenocarcinoma by upregulating T-cell checkpoint molecules^206^. BLZ945 inhibits glioma progression^207^ and delays cervical and mammary tumor growth^208^. Furthermore, eliminating interstitial macrophages by blocking CSF1R with the anti-CSF1R antibody CS7 prevents radiation pulmonary fibrosis^209^.

Furthermore, high-impact breakthroughs in the field of CSF1R inhibitors have identified the mechanisms by which CSF1R-expressing cells can promote the growth of cancer cells. In acute myeloid leukemia (AML), cells that express CSF1R support cancer cells by secreting hepatocyte growth factor (HGF) and other cytokines that help cancer cell survival and proliferation. Consequently, GW-2580 decreases the viability of AML patient samples, while resistance to GW-2580 is linked to decreased overall survival^210^. In mantle cell lymphoma (MCL), primary MCL cells transform monocytes into specific CD163+ M2-like macrophages by secreting CSF1 and IL-10. These macrophages, in turn, increase the survival and proliferation of MCL cells, and GW2580 reduces MCL cell viability in samples that are both resistant and nonresistant to the BTK inhibitor ibrutinib^211^. In chronic lymphocytic leukemia (CLL), CD14-positive cells that express CSF1R support CCL cell survival, and treatment with GW2580 or ARRY-382 decreases CCL cell viability^212^. In multiple myeloma (MM), blocking CSF1R with CS7 inhibits the proliferation and differentiation of M2 macrophages and myeloma-associated macrophages (MAMs). In the murine MM model, a CSF1R inhibitor demonstrates a therapeutic effect against cancer by repolarizing MAMs to adopt an M1-like phenotype and inducing a cytotoxic CD4+ T-cell response against tumor cells^213^.

However, the use of CSF1R inhibitors alone as a cancer treatment has also drawn conflicting conclusions. Single-cell RNA sequencing analysis reveals the presence two different TAM populations in colorectal cancer patients: tumor-suppressive TAMs, which have inflammatory gene signatures, and tumor-promoting polymorphonuclear myeloid-derived suppressor cells (PMN-MDSCs), which have an angiogenic gene signature^214^. Treatment with anti-CSF1R depletes only TAMs with an inflammatory gene signature but not PMN-MDSCs in colorectal cancer patients, leading to cancer-promoting effects through the accumulation of tumor-infiltrating PMN-MDSCs, which aggregated in tumors. In turn, simultaneous treatment with both a CSF1R inhibitor and a CXCR2 inhibitor targeting PMN-MDSCs effectively decreases tumor growth^215^. In two breast cancer models, CSF1 neutralization increases spontaneous metastasis without altering primary tumor growth in mice^216^. Targeting CSF1R by imatinib or AFS98 inhibits bone metastases in breast cancer by suppressing osteoclasts^217,218^. Another study also shows that simultaneous inhibition of CSF1R and SHP2 via dual-inhibitor-loaded nanoparticles (DNTs) is effective in mitigating tumors in breast cancer and melanoma mouse models^219^. Furthermore, dual inhibition of CSF1R and MAP kinase signaling also results in reduced tumor growth by reprogramming M2 macrophages to adopt the M1 phenotype^220^. The beneficial effects of targeting CSF1R, along with other therapeutic interventions, need to be further investigated.

### Clinical safety of inhibiting CSF1R

Although CSF1R deficiency in mice leads to significant defects in the functions of macrophages and osteoclasts, targeting CSF1R via small molecule inhibitors and monoclonal antibodies is relatively well tolerated and shows manageable adverse events. The dose-limiting toxicities of CSF1R inhibition have also been reported in only a few studies (reviewed in ref. ^221^). Several safety studies of CSF1R inhibition in healthy subjects, patients with rheumatoid arthritis, and patients with advanced or metastatic cancers, including giant cell tumors, glioblastoma, and Hodgkin lymphoma, have been reported^174,222–225^. Although there are some differences between small molecule inhibitors and monoclonal antibodies, the commonly reported adverse events for both inhibitors and antibodies are fatigue, nausea/vomiting, facial and peripheral edema, asthenia, pruritus, rash, headache, dry skin, increased lacrimation, and decreased appetite^226^. However, serious adverse events such as periorbital edema, lupus erythematosus, erythema, and dermo-hypodermatis have also been reported in trials using monoclonal antibodies to block CSF1R activation^227^. Nonfatal liver toxicity and elevated liver enzymes such as aspartate aminotransferase (ALT) and alanine transaminase (ALT) have been reported in clinical trials of CSF1R inhibition and may result from the depletion of CSF1R-expressing Kupffer cells from the liver or from targeting other receptors such as KIT. Although increased liver enzymes are short-lived or tolerated in most studies, a phase III study of pexidartinib was suspended due to fatal liver toxicity (NCT02371369). In clinical studies, the effect of CSF1R inhibition on bone health has not been well characterized. A FPA008 trial in 12 rheumatoid arthritis patients and a phase 1 study of ARRY-382 in cancer patients have been reported as meeting abstracts and show that CSF1R inhibition leads to increased serum CSF1 and/or IL-34 levels and diminished NTX^228,229^. How CSF1R inhibition affects osteoclast precursor cells and osteoclasts remains unknown, and the effect of CSF1R inhibition on bone remodeling needs to be further evaluated in human studies.

## Perspectives

Considerable research conducted over the past decades has identified the function of CSF1R-mediated signaling under physiological and pathological conditions. IL-34 and CSF1 are increased in pathological conditions and are considered potential biomarkers for disease prognosis and/or therapeutic targets. Over the past several years, the biological characteristics of IL‐34 and the importance of the IL‐34 signaling network in health and disease have been discovered. Although the binding of CSF1R with IL-34 and CSF1 activates similar signaling pathways, IL-34 and CSF1 have overlapping but distinct binding to CSF1R. IL-34 and CSF1 show functional differences and activate downstream signals with different intensities and differential targets. Therefore, understanding the differential mechanisms of CSF1- or IL-34-mediated regulation of osteoclastogenesis will be important, and CSF1R-targeting strategies in bone diseases may need to take into account the effects of alterations in IL-34-mediated signaling pathways.

Targeting CSF1R has gained much attention in many disease conditions in which myeloid cells play an important role. There have been several clinical trials targeting CSF1R or CSF1. However, recent clinical trials targeting CSF1R in inflammatory diseases or cancer have not been promising^161^. Emerging data address the potential mechanism by which CSF1R blockade has been ineffective in inflammation and cancer. CSF1R-expressing cells may play an anti-inflammatory role or a cancer-suppressive role. CSF1 might negatively regulate inflammatory responses by activating PI3K^230^, and CSF1R is expressed on both tumor-suppressing and tumor-promoting myeloid cells^215^. Thus, it is still necessary to clarify whether targeting CSF1R will be beneficial for clinical intervention in osteoclast-mediated pathological bone destruction, such as inflammatory bone destruction and bone osteolysis in tumors. Given that CSF1R plays a critical role in most myeloid lineage cells, a better understanding of how CSF1R regulates disease progression is critical for developing a specific therapeutic interventions or appropriate therapeutic strategies.
